# Music intervention on physiological and psychological responses of patients with cancer: A protocol for meta-analysis

**DOI:** 10.1097/MD.0000000000024865

**Published:** 2021-02-26

**Authors:** Nan Li, Dan Liu, Lei Zhang, Linshan Zhao, Fan Guo, Aimin Zang

**Affiliations:** a College of Clinical Medicine, Hebei University, Baoding, Hebei; b Beijing Longfu Hospital, No.18 Meishuguan East Street, Dongcheng District, Beijing; c Department of Medical Oncology, Affiliated Hospital of Hebei University, Hebei Key Laboratory of Cancer Radiotherapy and Chemotherapy, 212 Yuhua East Road, Baoding, Hebei, PR China.

**Keywords:** cancer, music intervention, outcomes, protocol

## Abstract

**Background:**

Cancer is a life-threatening condition and also one of the biggest challenges facing human health and the medical community. This meta-analysis was to investigate the effects of music intervention on physiological and psychological responses of patients with cancer.

**Methods and analysis:**

The following electronic databases will be searched from inception to December 2020: PubMed, EMBASE, Web of Science, Chinese Biomedical Literature Database, Chinese National Knowledge Infrastructure Database, Chinese Science and the Wanfang Database. We only included music intervention vs placebo in cancer patients and pooled results were summarized by STATA 12.0 software. Two investigators independently selected the studies according to the inclusion and exclusion criteria, extracted the data, and assessed the quality of the selected studies. The existence of statistical heterogeneity would be evaluated by Chi^2^ test and its extension by the *I*^2^ test (*I*^2^ > 50% indicates high heterogeneity among studies). Publication bias was ruled out by funnel plot and statistically assessed by Begg test (*P* > .05 as no publication bias).

**Results:**

The study results will be published in relevant peer-reviewed journals and key findings will be presented at international scientific meetings.

**Conclusion:**

Our study aims to systematically assess the effects of music intervention in cancer patients, which will be provide clinical guidance for cancer patients.

## 1 Introduction

Cancer is the second most common causative disease threatening human life.^[1]^ The World Health Organization (2009) reported that 7 million people worldwide died from cancer in 2005.^[2]^ Up to 35% of cancer patients may have mood, anxiety, or adjustment disorders.^[3]^ Cancer patients develop symptoms such as gait difficulty, dysarthria, dysphagia, and respiratory disorder, which confound their freedom and ability to communicate.^[4]^ These symptoms can seriously affect patients’ quality of life. Both qualitative and quantitative studies have shown depression and anxiety as the 2 most frequently reported psychological distress among cancer patients.^[5]^ Tumor patients have obvious psychological stress response or psychological disorders, 18% of the patients met the diagnosis of major depressive episode.^[6]^ Therefore, some countermeasures have been taken to circumvent the anxious mood of cancer patients.

Skills for managing negative emotional responses and distressful symptoms are critical to the quality of life of a patient with cancer.^[7]^ A variety of psychotherapeutic interventions, such as cognitive restructuring, relaxation training, guided imagery and music, have shown promise in decreasing psychological distress during cancer treatments.^[8]^ Music therapy as a clinical psychotherapy model has been applied to a broad spectrum of populations in the health system.^[9]^ Music therapy was based on psychotherapy theory and aimed to remove psychological barriers and restore physical and mental health.^[10]^ Nevertheless, the effect of music therapy in improving physiological and psychological responses has not been elucidated.

In this meta-analysis, we systematically reviewed relevant published articles about music intervention for cancer patients to analyses the effectiveness of music intervention physiological and psychological responses in cancer patients.

## 2 Methods and analysis

### 2.1 Participants

#### 2.1.1 Study registration

The Preferred Reporting Items for Systematic Reviews and Meta-Analysis Statement was applied for guiding this systematic review and meta-analysis.^[11]^ This meta-analysis was registered in the INPLASY (INPLASY202110082). And this study protocol was funded through a protocol registry. This study receives ethics approval from Affiliated Hospital of Hebei University and founded by Youth scientific research foundation of Affiliated Hospital of Hebei University (2017Q002).

### 2.2 Inclusion and exclusion criteria

The details regarding the inclusion criteria are listed as follows:

1. Patients: cancer patients aged 18 to 75 years;
2. Intervention: music intervention;
3. Control: placebo;
4. Outcomes: state-trait anxiety inventory, self-rating anxiety scale, self-rating depression scale and visual analogue scale; and
5. Study design: randomized controlled trials.

The study selection process will be carried out in 2 stages (screening of title/abstract, and full-text assessment of articles), with 2 reviewers independently, and in duplicate, determining inclusion/exclusion of study records based on previously specified criteria.

### 2.3 Study search

The following electronic databases will be searched from inception to December 2020: PubMed, EMBASE, Web of Science, Chinese Biomedical Literature Database, Chinese National Knowledge Infrastructure Database, Chinese Science and the Wanfang Database, and using the following core terms: (“cancer” OR “tumor” OR “Neoplasms” OR “Neoplasias” OR “Malignant Neoplasm”) AND (“music intervention” OR “music therapy”). The studies have already completed but not yet published were searched from the website http://clinicaltrials.gov/ (US NIH) and the Meta Register of Controlled Trials. We also reviewed the reference lists of identified studies to identify any new RCT met the inclusion criteria.

### 2.4 Study selection

**Figure 1:**
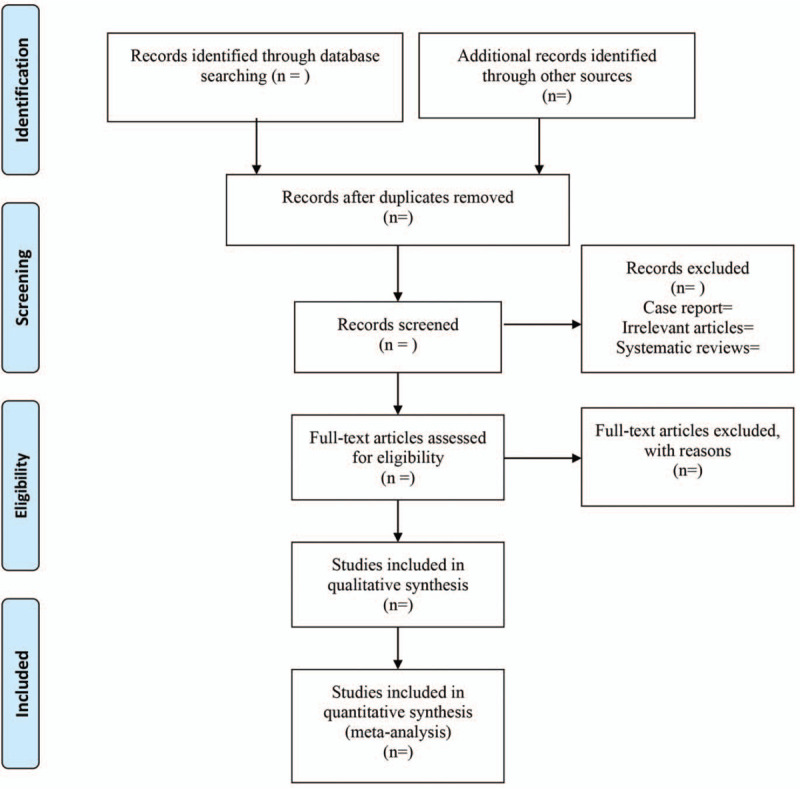
The flow diagram of procedure to select studies.

Two independent investigators screened title, abstract, and full content of articles, with inconsistencies resolved through discussion between 2 examiners. If consensus was not reached, then final conclusion was reached by an independent expert provided advice. The study flow chart is presented in Figure 1.

### 2.5 Data extraction

The following information were abstracted by 2 reviewers independently: first author name, publication year, country, sample size, percentage male, mean age, type of operation, anesthesia, intervention, control, and investigated outcomes (state-trait anxiety inventory, self-rating anxiety scale, self-rating depression scale and visual analogue scale), any disagreement between reviewers was settled by discussion until a consensus.

### 2.6 Risk of bias assessment

Two examiners (Nan Li and Dan Liu) independently in blind performed quality and risk of bias assessment. The third expert would join in to discuss and resolve divergence. Risk of bias assessment was assessed using the Cochrane Collaboration's tool for assessing risk of bias in randomized trials. Because the number of studies included in this meta-analysis of randomized, double or triple-blind, controlled clinical trials was 8 less than 10, the funnel plot was not used to explore publication bias.

### 2.7 Data analysis

Review Manager software (version 5.3; Nordic Cochrane Centre, The Cochrane Collaboration,) was used to perform data synthesis and analysis. For dichotomous outcomes, that is adverse events, risk ratios with corresponding 95% confidence intervals (CIs) was used to estimate pool effects. For continuous outcomes, when included studies not directly reported data used to aggregate effects.^[12]^ These data were calculated from 95% CIs and changes from baseline to 6, 12 months. Further, when there were more than 2 intervention groups or control groups in an included trial, we joined 2 groups into a single group. We used mean differences and 95% CIs for outcomes with the same measures; standard mean differences (SMDs) and 95% CIs for outcomes with different measures. A SMDs of 0.20 is considered a small difference between the experimental and the control group; 0.50, a moderate difference; and 0.80, a large difference. Statistical heterogeneity among included studies was assessed with *I*^2^ statistic: 0% to 40%, might not be important; 30% to 60%, may represent moderate heterogeneity; 50% to 90%, represent substantial heterogeneity; 75% to 100%: considerable heterogeneity. As heterogeneity in risk ratios, mean differences, SMDs is 0% or might not be important, fixed-effect 176 was used to pool the results. All statistical tests undertaken were 2-sided and considered a *P* value ≤.05 or a 95% CIs that excluded a null result as statistically significant.

## 3 Discussion

The purpose of this meta-analysis was to summarize the existing evidences about music intervention in improving physiological and psychological responses in cancer patients. This study has some highlights. First, this is the first systematic review and meta-analysis about the effectiveness of music intervention in cancer patients. In addition, we systematically searched the both English and Chinese databases to comprehensively selected the published papers. Moreover, we performed subgroup analysis and sensitivity analysis to increase the reliability of our meta-analysis. Publication bias was finally performed to identify the potential publication bias between the included studies. Finally, we could provide evidence for clinical guidance of cancer patients.

## Acknowledgment

We would thank for the INPLASY platform for registry for this study.

## Author contributions

**Data curation:** Nan Li.

**Formal analysis:** Nan Li.

**Investigation:** Lei Zhang.

**Project administration:** Lei Zhang.

**Supervision:** Aimin Zang.

**Validation:** Aimin Zang.

**Visualization:** Dan Liu, Linshan Zhao, Fan Guo.

**Writing – original draft:** Dan Liu, Linshan Zhao, Fan Guo.
